# Pathologic Conditions of the Pharynx and Contiguous Structures during Early Childhood—Prime Factors in the Etiology of Mal-Formed Maxillæ and Irregular Teeth, Etc.

**Published:** 1897-08

**Authors:** W. A. Mills

**Affiliations:** Baltimore, Md.


### Pathologic Conditions of the Pharynx and Contiguous Structures During Early Childhood—Prime Factors in the Etiology of Mal-Formed Maxillæ and Irregular Teeth, Etc.

BY W. A. MILLS, D.D.S., BALTIMORE, MD.

Abstract of a paper read before the Dental Section, American Medical Association, Philadelphia, June, 1897.

The physical functions of the nasal cavities are, viz: They elevate the temperature of the inhaled air, give it moisture and purify it, by arresting all particles of dust and other substances which it may contain; they act as resonance cavities for the voice, and are also the seat of the sense of smell. A professional experience of more than twenty-five years has taught us that any inflammatory lesions found in children between the ages of four and twelve, which obstruct these natural conditions, are, in the majority of cases, the chief agents in the causation of mal-formed jaws and the consequent abnormal alignment of the teeth. We cite the following case in our practice:

A boy, age seven years, was brought to the office to have a tooth filled, which was supposed to cause pain in the right ear and right angle of the inferior maxillæ. There was almost complete obstruction of the nasal cavities, face pale, anemic and haggard-looking features, contracted or pinched at the angle of the jaw, and beneath the malar processes, giving the characteristic of a mouth-breather.

On examination we found a small cavity in a molar tooth, which in no manner could have caused the pain. This was filled but gave no relief. The tonsils were greatly enlarged, almost meeting at the median line. Deglutition was both difficult and painful. On examining the superior maxillæ, we noticed a very perceptible contraction on both sides, anterior to the first permanent molar teeth, with a decided inclination of the roof of the mouth to become elevated. We informed the patient's aunt of the state of affairs with the assertion that unless something heroic was done quickly, and by a rhinologist, very serious consequences would be sure to follow.

Six months later the patient returned to the office. A specialist had been consulted, and he had removed some adenoid growths and was still reducing the tonsils with the galvano-cautery. We found a bright and rosy-faced boy with normal respiration, and imagine our surprise when upon examination we found no signs of the former contractions of the jaw or rising of the arch, but all outlines were normal.

This particular case demonstrated that either the enlarged and diseased tonsils, or the adenoid vegetations, or both, were the prime factors in the causation not only of the mal-formations of the maxillæ, but also the oral respiration. If a correct diagnosis had not been made, and only palliative treatment been given, there is no doubt that a case of V or saddle-shaped arch would have been developed with irregular teeth as concomitants, especially so as the patient's parents are full-blooded Germans and have large jaws and large teeth.

The starting point in the majority of these cases has its origin in some slight irritation of the mucous membrane of the throat, especially the tonsils.

We would urge upon the medical and dental practitioners the importance of being ever on the alert to discover the first signs of any inflammatory manifestation in the throat or nasal cavities of children, as so many lesions are prone to develop along the lines of the respiratory passages, without in themselves showing any very acute symptoms; and if permitted to continue without proper treatment, cause disease and mal-formation, which in some cases defy all remedial efforts.

Dr. M. H. Cayer, says: "It is my opinion that an inflammation of the tonsil and surrounding tissue will cause tension of the palato-pharyngeal and palato-glossus muscles; if this be so they would naturally pull the lateral portions of the arch downward and inward; especially is this the case with children when their bones are soft and yielding.

